# Heart rate variability in chronic ischemic stroke: analysis during the sleep-wake cycle

**DOI:** 10.1055/s-0044-1791660

**Published:** 2024-11-06

**Authors:** Natalia Buitrago-Ricaurte, Fatima Dumas Cintra, Jean Faber, Gisele Sampaio Silva

**Affiliations:** 1 Universidade Federal de São Paulo, São Paulo SP, Brazil.; 2 Universidad del Rosario, Bogotá, Colombia.; 3 Universidade Federal de São Paulo, Departamento de Cardiologia, São Paulo SP, Brazil.; 4 Universidade Federal de São Paulo, Departamento de Neurologia e Neurocirurgia, São Paulo SP, Brazil.; 5 Hospital Albert Einstein, São Paulo SP, Brazil.

**Keywords:** Ischemic Stroke, Heart Rate Determination, Autonomic Nervous System, Stroke, Circadian Rhythm, Acidente Vascular Cerebral Isquêmico, Determinação da Frequência Cardíaca, Sistema Nervoso Autônomo, Acidente Vascular Cerebral, Ritmo Circadiano

## Abstract

**Background** Alterations of the autonomic nervous system (ANS) in the chronic stage of ischemic stroke (IS) are not well understood. Heart rate variability (HRV) provides a noninvasive approach to assess autonomic function.

**Objective** To compare the HRV parameters during the sleep-wake cycle between patients with IS in the chronic stage and healthy subjects.

**Methods** We conducted a retrospective transversal study based on clinical records and 24-hour electrocardiogram (EKG) monitoring registries of 179 patients with a confirmed IS diagnosis and 184 age- and sex-matched healthy subjects. Circadian variation was calculated according to the variation of the total autonomic activity (VTAI) and the parasympathetic activity (VPAI) indexes. Comparisons were performed using nonparametric tests. Multivariable analyses were performed with canonical discriminant analysis (CDA) and a three-way analysis of variance (ANOVA). Statistical significance was established with a confidence level of 95%.

**Results** During waking hours, the healthy group exhibited higher variability in the time domain and frequency domain parameters: standard deviation of NN intervals (SDNN, *p* < 0.001) and of the average NN intervals (SDANN, *p* < 0.001), as well as low-frequency (LF) band ( *p* < 0.001). During sleep, the difference was higher in the high-frequency (HF) band ( *p* < 0.001), and lower in the low-/high-frequency ratio (LF/HF, *p* < 0.001). Both VPAI and VTAI showed less significant difference in IS patients ( *p* < 0.001).

**Conclusion** There was diminished heart vagal activity among IS patients, as measured through HRV. During sleep, this is likely caused by an imbalance in the sympathetic and parasympathetic systems shifting through the sleep phases. These imbalances could persist over time in patients with IS, lasting months after the initial injury.

## Resumo

**Antecedentes** A compreensão das alterações do sistema nervoso autônomo (SNA) na fase crônica do acidente vascular cerebral isquêmico (AVCi) ainda é insuficiente. A variabilidade da frequência cardíaca (VFC) fornece uma abordagem não invasiva para avaliar a função autonômica.

**Objetivo** Comparar os parâmetros da VFC durante o ciclo sono-vigília entre participantes saudáveis e com AVCi crônico.

**Métodos** Estudo retrospectivo transversal baseado em registros clínicos e registros de eletrocardiograma de 24 horas de 179 pacientes com AVCi confirmado e 184 controles pareados por idade e sexo. As variações do índice de atividade autonômica total (VTAI) e do índice de atividade parassimpática (VPAI) foram usados para calcular a variação circadiana. As comparações foram feitas usando testes não paramétricos. Análise discriminante canônica (CDA) e análise de variância (ANOVA) de três vias foram usadas para análises multivariadas. Um nível de confiança de 95% foi estabelecido para a significância estatística.

**Resultados** O grupo controle exibiu maior variabilidade nos parâmetros de domínio do tempo e da frequência durante as horas de vigília: desvio padrão dos intervalos NN (SDNN, *p* < 0,001) e das médias dos intervalos NN (SDANN, *p* < 0,001), assim como a banda de baixa frequência (LF, *p* < 0,001). Durante o sono, a diferença foi maior na banda de alta frequência (HF, *p* < 0,001) e a razão baixa/alta frequência (LF/HF) foi menor ( *p* < 0,001). O VPAI e VTAI mostraram menor variabilidade nos pacientes com AVC ( *p* < 0,001).

**Conclusão** Os pacientes com AVCi apresentaram atividade vagal cardíaca diminuída, medida pela VFC. Isto pode resultar de desequilíbrios nos sistemas simpático e parassimpático que mudam durante as fases do sono. Em pacientes com AVCi, esses desequilíbrios podem permanecer meses após a lesão inicial.

## INTRODUCTION

Ischemic stroke is a highly prevalent disease with an increasing prevalence and decreasing mortality rate worldwide. 1 More than 40% of patients with stroke have sequelae affecting multiple organ systems, with long-term consequences. 2 The central autonomic network (CAN) injuries after IS could relate to poststroke autonomic dysfunction and other stroke complications 3 that impact patients' risk of recurrence and recovery status. 4 5

An accessible, noninvasive, widespread approach for studying autonomic function after stroke is the heart rate variability (HRV) analysis, 6 that evaluates the interaction of multiple structures and autonomic relay stations. Because of technical difficulties in assessing the CAN function, HRV analysis estimates the effector responses of the ANS in the cardiovascular system. 7 This method focuses on the response of the heart as an effector organ of the ANS, measured by the surface electrical activity of the heart and visualized in an electrocardiographic (EKG) record spanning from 20 minutes to 24 hours or more. 8 The HRV parameters also allow for an effective analysis of the parasympathetic and sympathetic nervous systems' activity in different diseases, as well as in different physiological instances, such as in the circadian rhythm. 9

Changes in HRV among IS patients during the acute stage are well described in the literature and relate to cardiac and infectious complications, which are leading nonneurological causes of death. 10 11 12 Evidence of autonomic function during the chronic stage of IS is limited, but some studies have shown a persisting sympathetic and parasympathetic activity imbalance during this later stage of the disease. The HRV analysis could be a useful tool for studying poststroke autonomic dysfunction and proposing underlying mechanisms. 13 However, there are few studies available regarding HRV in ischemic stroke patients, mainly focused on the acute stage with small samples that did not consider other variables, such as medication use, etiology, associated risk factors, and circadian variation. 14 15

Our study evaluated the HRV parameters of IS patients in the chronic stage during the sleep-wake cycle. We aimed to associate variables like sex, age, classical cardiovascular risk factors, stroke etiology, topography, functionality scales, and variation in the sleep-wake cycle with the hypothesis that ischemic stroke subjects exhibit altered HRV parameter dynamics. Therefore, the present study attempts to describe the HRV parameters during the sleep-wake cycle in patients with IS and compare them to healthy subjects. Our results will contribute to the body of evidence on the behavior of these parameters in our population, as well as propose future research targets.

## METHODS

We performed a retrospective, transversal study at the Hospital de São Paulo. Once the research ethics committee approved the protocol (study:1383/2018, approval: 3.129.451), a total of 179 patients with previous ischemic stroke and 184 age- and sex-matched healthy subjects from the cardiology department of our hospital gave authorization and written consent for their inclusion in the study.

**Figure 1:**
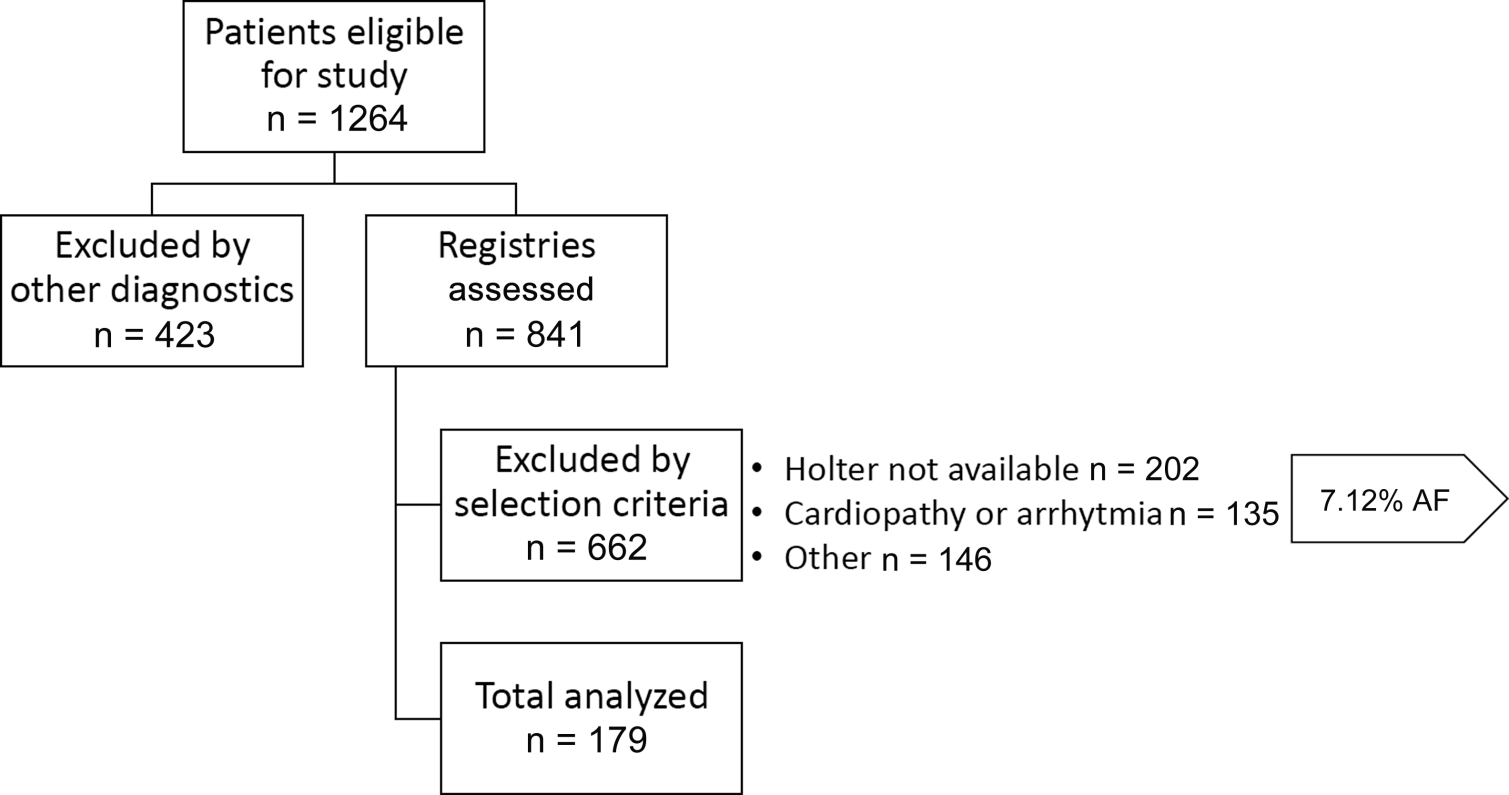
Flowchart of selected patients.

Subjects were selected according to the inclusion criteria ( Figure 1 ) and sample size recommendations for HRV retrospective studies. 16 The clinical records and Holter registries of patients between 2016 and 2019 were analyzed. They were admitted 7 months after IS (interquartile range [IQR]: 9 months) and monitored in the outpatient setting recorder for 24 hours with the CardioSmart Institutional CS 550 recorder (Cardio Sistema Comércio e Indústria Ltda, São Paulo, SP, Brazil), and all being analyzed by the same software to obtain time- and frequency-domain parameters.

The recordings were purified for artifacts by a specialized technician. Each QRS complex was detected, and the intervals between two normal QRS complexes (NN intervals) were selected based on a cubic spline interpolation filter with a maximal correction factor of 20%. Four time-domain indexes were obtained: the standard deviation SD) of all NN intervals (SDNN), the SD of the average NN intervals (SDANN), the root mean square of the difference between consecutive NN intervals (RMSS), and the proportion of adjacent normal NN intervals differing by more than 50 ms (pNN50).

For frequency domain analysis, artifact-free recordings were computed by fast Fourier transformations using 5 minutes Blackman-Harris windows (12 segments per hour, points per frequency domain 300 points/Hz, interpolation rate 4Hz). The power densities were obtained by integrating the power spectral density in each frequency band: high frequency (HF: 0.15–0.5 Hz), low frequency (LF: 0.04–0.15Hz), and very low frequency (VLF: 0.0033–0.04 Hz). 17 The results were expressed in normalized units. Also, the LF/HF index was calculated and expressed in a normalized power spectrum. 18 19

The wake and sleep measurements were based on subjects' journal entries. For the sleep measurements, the selection was based on the predominant parasympathetic impulse measured by the HF variable during the 90 to 120 minutes after the subject fell asleep. 20

Sympathetic and parasympathetic activity indexes evaluated sleep-wake variation. The former was previously described for evaluating variations of sympathetic activity (VSAI). 21 Recognizing that the LF parameter is not solely the result of sympathetic influx, 22 we dubbed it variations of the total autonomic activity index (VTAI), a parameter that could aid in comparing vagal activity to total autonomic activity over time.

The variation of the parasympathetic activity index (VPAI) was created as an approach to assess parasympathetic variations based on that index. It is important to note that this is not a validated index and that further studies are required to understand its significance and clinical applicability.

We reviewed all digital records of patients followed in our stroke clinics from 2016 to 2019 to select them by purposive sampling according to the inclusion criteria. Patients with confirmed ischemic stroke, age greater or equal to 18 years, Holter in the 12 months after stroke onset, clinical record with complete information, nonrecurrent or multiple topographies ischemic stroke, and absence of conditions that may affect HRV measures such as heart failure with reduced ejection fraction, atrial fibrillation or other arrhythmias, pacemaker use, and known dysautonomic disorder were included. All data were obtained directly from the neurovascular outpatient visit recorded in the medical chart following the process shown in Figure 1 . We obtained 14 variables: sex, age, race, presence of hypertension, diabetes, dyslipidemia, use of tobacco, used medication, as well as different stroke topographies, them being laterality, localization and TOAST classification, NIHSS during the event, NIHSS at the moment of clinical evaluation, and modified Rankin score. If NIHSS was not described in the chart, we calculated it based on the previously stated methodology. 23

### Statistical analysis

Descriptive statistics were applied for all the analyzed variables and the Kolmogorov-Smirnoff normality test was performed. The Chi-squared test was used for categorical variables. Bivariate comparisons were performed using the Wilcoxon signed-rank test for paired samples and the Wilcoxon rank-sum test for nonpaired samples. For bivariate analysis between groups, the Kruskal-Wallis test was performed. Cliff delta was used as a nonparametric effect size measure due to multiple comparisons.

For the multivariate analysis, the three-way analysis of variance (ANOVA) was used for interest variables adjusted for sex and age. Canonical discriminant analysis (CDA) was performed to analyze the variables' distribution patterns between and inside the groups to make them more consistent. The statistical significance was established with a confidence level of 95%. The CDA technique approach optimizes the separation between the possible groups observed in an independent variable X related to the labels described by the categorical dependent variable Y. 24 All the factors described in X have the same weight concerning the assignment of the interest groups. The CDA also permits the establishment of relationships among classes defined by the categorical variable Y and the set of variables described in X. Here, we considered HF, LF, HF/LF, and VLF as the main features for X, for the 362 patients in each one of the four classes: CTRL:Wake (healthy subjects-wake parameters), CTRL:Sleep (healthy subjects-sleep parameters), Stroke:Wake (Stroke patients-wake parameters), Stroke:Sleep (Stroke patients-sleep parameters). It means that Y is labeled by these four groups. In this manner, X is a matrix of (4 classes × 362 patients) × 4 features and Y is a vector of (4 classes × 362 patients).

Therefore, if the four chosen features have enough information to discriminate each group described by Y, the CDA will be able to identify them. Moreover, we provided some criteria such as “IS patients / Healthy subjects,” “Sleep/Wake,” and “Parasympathetic/Sympathetic,” and the technique identified if they have distinct characteristics, showing it in distribution patterns as clusters. The Matlab R2019a (MathWorks Inc., Natick, MA, USA) statistics and machine learning toolbox was used for statistical analysis.

## RESULTS

A total of 1,264 clinical records were revised, 841 of which were eligible based on our inclusion criteria. From these, 483 were excluded due to other conditions (hemorrhagic stroke, transient ischemic attack, aneurysms, subarachnoid hemorrhage), resulting in a final sample of 179 patients (43% females, mean age: 63 years, IQR: 53.65–71.37). The control group comprised 184 sex and age-matched healthy adults (44% females, mean age: 63 years, IQR: 53.65–71.37).

### Descriptive analysis

The most frequently associated stroke risk factors were hypertension (78%), smoking (53%), dyslipidemia (53%), and diabetes (34%). The most prescribed medications were aspirin and antihypertensives. Healthy subjects did not report any regular medication use, but smoking was found in 19.56% ( Table 1 ).

**Table 1:** Baseline characteristics

		IS patients ( *n* = 179)	Healthy subjects ( *n* = 183)	*p* -value
Age (IQR)		63 (53.65–71.37)	63 (53.62–71.39)	0.96
Women n (%)		77 (43%)	82 (44.08%)	0.82*
Comorbidities	HBP n (%)	139 (77.6%)	NA	
	Smoking n (%)	94 (52.5%)	36 (20.11%)	< 0.001*
	DLP n (%)	95 (53%)	NR	
	DM-2 n (%)	61 (34%)	NR	
Medications	AAS n (%)	120 (67%)	NA	
	ECA n (%)	53 (29%)	NA	
	ARB n (%)	53 (29%)	NA	
	CAA n (%)	57 (31.84%)	NA	
	BB n (%)	45 (25.13%)	NA	
	NONE n (%)	4 (2.23%)	NA	

Abbreviations: AAS, Acetylsalicylic acid; ARB, Angiotensin II receptor blocker; BB, β-blocker; CAA, calcium antagonist; DLP, dyslipidemia; DM-2, diabetes mellitus type 2; ECA, angiotensin-converter enzyme blocker; HBP, high blood pressure; IQR, interquartile range; IS, ischemic stroke; n, number of subjects; NA, not available.

Note: *χ ^2^ test with a significance level of 95%.

Stroke topography was predominantly on the left side (52.51%) and in the anterior circulation, with cortical (41.3%), insular cortex (16.2%), and subcortical (17.8%) lesions. In the posterior circulation, the lesions were cerebellar (8.7%) and in the brainstem (16.2%). The etiology of the event was defined as cryptogenic in 55% of the patients, with large vessel atherosclerosis in 15.6%, cardiogenic in 13.4%, small vessel disease in 11.73%, and other causes in 4% of the patients. The median NIHSS calculated by the ER department during the acute stage was 6 points (IQR: 6 ± 3) and the median score during the second visit to the stroke clinic was 3 points (IQR: 3 ± 2). Also, the median modified Rankin score calculated during the second visit was 2 points (IQR: 1 ± 2).

The frequency of cardiovascular risk factors was different among patients with different IS etiologies, according to the TOAST classification. Hypertension was more common in patients with small vessel, cryptogenic, and cardioembolic strokes ( *p* < 0.001). Patients with stroke etiology classified as “other causes” exhibited a lower frequency of classical cardiovascular risk factors than those in the etiological categories. There were no differences in the analyzed parameters between the subacute and chronic stages.

### Analysis between groups

There were no differences between the two groups in the minimal, average, or maximal heart rates per subject during the 24 hours. In the time-domain analysis, the SDNN and SDANN were higher in the healthy subjects. In the frequency-domain analysis of the HRV, for the wake measurements, only LF was different (higher) in healthy subjects. In the nonREM measurements, HF was higher and LF/HF lower in the control group. The VPAI was higher in healthy subjects, a finding consistent with the discriminant component analysis of the frequency domain variables during awake and sleeping measurements. Finally, supraventricular and ventricular arrhythmias were more frequent in patients with stroke ( Table 2 ).

**Table 2:** Heart rate variability parameters comparison between patients and healthy subjects

		IS patients	Healthy subjects	*p* -value
**Minimum HR**		54 (11)	51 (10)	0.09
**Average HR**		76 (14)	74 (15)	0.08
**Maximal HR**		122 (24)	121 (24)	0.94
**Time-domain**	SDNN *(ms)*	84.1 (69)	111 (37)	**< 0.001♦**
	SDANN *(ms)*	84 (62)	97 (37)	**< 0.001♦**
	PNN50 *(%)*	2.97 (7)	3.14 (7)	0.65
	RMSS *(ms)*	22 (18)	25 (17)	0.06
**Frequency domain**	HFwake *(n.u)*	0.09 (0.18)	0.11 (0.13)	0.85
	LFwake *(n.u)*	0.25 (0.19)	0.28 (0.19)	**< 0.001♦**
	LF/HFwake	2.28 (3.5)	2.90 (4.3)	0.12
	VLFwake *(n.u)*	0.59 (0.34)	0.53 (0.32)	0.16
	HFsleep *(n.u)*	0.15 (0.27)	0.31 (0.31)	**< 0.001♦**
	LFsleep *(n.u)*	0.23 (0.17)	0.22 (0.17)	0.91
	LF/HFsleep	1.42 (2.5)	0.79 (1.43)	**< 0.001♦**
	VLFsleep *(n.u)*	0.54 (0.39)	0.40 (0.37)	**< 0.001♦**
**Indexes**	VPAI	0.023 (0.20)	0.16 (0.32)	**< 0.001♦**
	VTAI	0.001 (0.26)	-0.07 (0.25)	**< 0.001♦**
	Supraventricular (Mdn)	29 (195)	0 (30)	**< 0.001♦**
	Ventricular (Mdn)	32 (170)	3 (27)	**< 0.001♦**

Abbreviations: %, percentage; HF, high frequency; HR, heart rate; IS, ischemic stroke; LF, low frequency; Mdn, median; ms, milliseconds; n, number of subjects; n.u, normal units; PNN50, percentage of successive RR-interval differences > 50 ms; RMSS, the square root of the mean of the sum of squares of differences between adjacent RR-intervals; SDANN, standard deviation of the averages of normal RR intervals; SDNN, standard deviation of all normal RR intervals; VLF, very low frequency; VPAI, variation in parasympathetic activity index; VTAI, variation in total autonomic activity index.

Note: All variables are described in terms of median and interquartile range.

♦Mann-Whitney U (Wilcoxon rank-sum) bicaudal with a significance level of 95%.

### Analysis of variations during wake and sleep

The frequency-domain parameters for patients and healthy subjects behaved differently during the sleep-wake cycle for each group. The HF variation was higher in control patients than in stroke patients (Cliff δ: -0.34). Similarly, the LF/HF decreased more during sleep in healthy subjects than in IS patients (Cliff δ: 0.45).

No differences were found between IS patients and healthy subjects for the VLF measurements in the sleep-wake cycle.

The three-way ANOVA for adjusted analysis did not reveal differences between IS groups concerning age and sex. An analysis adjusted for age, topography, and etiology found that elderly patients with small vessel insular stroke had less significant variance in the parasympathetic variables. The VLF measurements were different for right and left basal nuclei strokes. Elderly patients with bilateral stroke showed less HF, RMSS, and pNN50 variation.

### Canonical discrimination analysis results

**Figure 2:**
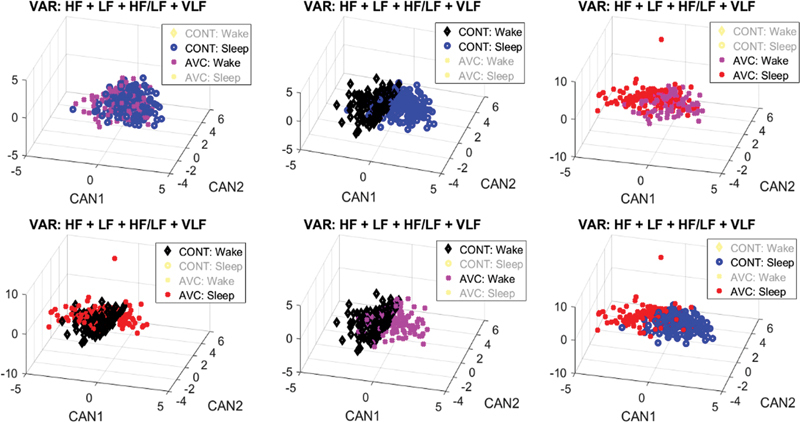
Canonical Discriminant Analysis (CDA) for the frequency domain variables for ischemic stroke (IS) patients and healthy subjects (HS). Healthy subjects showed different patterns of frequency-domain variables during wake and sleep (2A). In contrast, IS patients showed less variation in that variable during wake and sleep (2B). Comparing the groups, they had different patterns during wake (2C) an effect that is more evident during sleep (2D). Note: *CAN1 and CAN2 mean, respectively, the canonical variables 1 and 2 from the CDA analysis (see main text).

In the analysis of the frequency domain variables for IS patients and healthy subjects, different patterns were identified shown in Figure 2 , with canonical discriminant analysis used for the frequency domain variables for IS patients and healthy subjects). Also, the IS patients did not present a specific pattern for the distribution of variables during the sleep-wake cycle and, as a result, clusters could not be identified. Healthy subjects exhibited different patterns in the frequency domain for the wake and sleep variables, which resulted in the conformation of sleep and wake clusters, respectively. Therefore, this method suggests that the frequency domain variables in IS patients and healthy subjects have different distribution patterns, especially during sleep, which is similar to the nonparametric tests explained before ( Table 2 ).

## DISCUSSION

Our study showed that autonomic system imbalances assessed with HRV analysis are present in the chronic stages of IS. Because multiple factors such as stroke characteristics, comorbidities, and functional status (stroke severity, etiology classification, modified Rankin score > 3, prolonged-severe disability) can alter HRV parameters, we applied a strict selection criteria and a stratified analysis with different statistics methods to avoid confounding factors and possible bias. 25 We used an accessible tool, the prolonged EKG recordings to be able to evaluate the autonomic response along the sleep-wake cycle. In our patients during wakefulness, the time domain SDNN, SDANN parameters, and LF measures showed less variability. During sleep, IS patients showed less vagal activity as measured by HF and VPAI. Additionally, the LF/HF index was higher in these patients due to reduced HF activity; in the same manner, the VTAI was lower in IS patients, likely due to the reduced vagal contribution in the LF domain.

The ANS is a highly complex system that controls different biological networks with numerous relay and control stations regulating responses and processes for maintaining body homeostasis in different set points that vary in response to dynamic internal and external demands. 26 27 Therefore, studying autonomic dysfunction is challenging due to the systems' complexity and dynamic response changes in short periods of time, leading to a great variety of study methods. 28 The HRV is a very useful approach for studying ANS dynamics, seldom used in IS patients. 13 29 However, its evaluation in IS patients must be cautious, since factors that could interfere with its interpretation such as comorbidities like diabetes mellitus, arrhythmias, sleep disorders, medication use, and functional status are to be carefully considered.

Autonomic imbalances in IS patients are related to frequent complications in the acute stage of the disease. 12 27 30 These imbalances can be due to autonomic dysfunction or adequate autonomic responses to changing set points. 31 32 Autonomic imbalances in the heart's conduction pathways, for example, are associated with rhythm alterations, which, in conjunction with other factors (hypertension, sleep disorders, ischemic cardiopathy, diabetes mellitus), can result in structural tissue remodeling and trigger arrhythmogenic mechanisms. 33 34 Hence, changes in the ANS could have a causal association with the pathogenesis of IS, and their chronic persistence might indicate a higher risk of disease recurrence. 35 36

Studies that specifically evaluated HRV in patients with IS used heterogeneous protocols concerning recording, window selection, techniques employed for frequency analysis (AR, FFT, trigonometric regressive spectral TRS), and autonomic challenge maneuvers (Valsalva, deep breathing, handgrip). Conversely, linear HRV parameters are widely used but understanding HR control as a complex and nonlinear process requires understanding these linear parameters' role in the underlying mechanisms that mediate HR control. 37 38 Since prolonged electrocardiographic measurement is an accessible and frequently used method in clinical practice for the etiological investigation of IS, we estimate HRV could, through the implementation of diagnostic algorithms and AI technologies, become a valuable tool for understanding the implication of breathing and sleep cycles in the evolution of disease guidelines. Furthermore, protocols for HRV measurements in an IS context are required according to recent advances in research trends. 39

It is worth noting that, in our study, most of the selected patients were those who underwent a comprehensive etiological evaluation without identifying a specific cause for their stroke. Our patients exhibited the classical cardiovascular risk factors described in previous studies. Due to our selection criteria, we had more patients with undetermined stroke than other series in Latin America. 40 The frequency of cardiovascular risk factors was higher compared to populations with similar profiles. 41 Additionally, we excluded atrial fibrillation and heart failure, which are comorbidities that could affect cardiac autonomic activity. Therefore, it is recommended that researchers carefully analyze our results in other populations with IS.

The HRV alterations in patients with ischemic stroke could have a prognostic value for mortality and stroke recurrence, as well as for understanding etiology with consequent improvement in therapeutic strategies. Our findings suggest that stroke itself might alter the physiological effects of age and sex in HRV parameters, which is similar to previous studies' results. 15 42 43 44 Interestingly, we observed that supraventricular and ventricular extrasystoles were more frequent in IS patients, a finding that was not described in previous studies. A combination of cardiac morphology parameters, such as left atrium size and ejection fraction, with the linear and nonlinear HRV parameters could be a promising instrument to identify patients at high risk for stroke recurrence, improving diagnostic strategies and therapeutic options. 36 45

Our study has some limitations. While sleep disturbances, common in IS patients, are associated with cardiovascular risk factors and with atrial fibrillation, unfortunately, the frequency of sleep disturbances in the group of IS patients was unknown. Given that HRV parameters are generally altered in patients with sleep disorders, these disturbances are interaction factors to be considered for future studies. 46 47 Additionally, HRV parameters are correlated to other cardiovascular responses that can explain HR variations in more detail, like arterial blood pressure and respiration rate. Ideally, these variables should be measured along with the EKG recording. Unfortunately, the retrospective nature of our study did not allow for such correlations.

In conclusion, cardiac autonomic disturbances in ischemic stroke patients could persist in the chronic stage of the disease. Our results suggest that HRV analyses in the time and frequency domains differed in ischemic stroke patients when compared with healthy subjects matched for age and sex. During wakefulness, IS patients showed less variability and expression of vagal activity, an effect that is more pronounced in IS patients during the sleep cycle, when all variables that measure it are lower.
